# Effects of Nandrolone Decanoate on Skeletal Muscle and Neuromuscular Junction of Sedentary and Exercised Rats

**DOI:** 10.3390/medicina59111940

**Published:** 2023-11-01

**Authors:** Felipe Cantore Tibúrcio, Ana Paula Silveira Leite, Kevin Silva Muller, Carina Guidi Pinto, Erick Valentino, Paula Aiello Tomé de Souza Castro, Cintia Yuri Matsumura, Shelly Favorito de Carvalho, Selma Maria Michelin Matheus

**Affiliations:** 1 Medical School, São Paulo State University (Unesp), Botucatu 18618-687, SP, Brazil; felipe.cantore@unesp.br (F.C.T.); ana.p.leite@unesp.br (A.P.S.L.); kevin.muller@unesp.br (K.S.M.); carina.guidi@unesp.br (C.G.P.); 2 Division of Anatomy, Department of Structural and Functional Biology, Institute of Biosciences, São Paulo State University (Unesp), Botucatu 18618-689, SP, Brazil; cintia.matsumura@unesp.br (C.Y.M.); 3 Department of Physical Therapy, Center for Biological and Health Sciences, Federal University of São Carlos (UFSCar), São Carlos 13565-905, SP, Brazil; paula.castro@ufscar.br; 4 Electron Microscopy Center, Institute of Biosciences, São Paulo State University (Unesp), Botucatu 18618-689, SP, Brazil; shelly.favorito@unesp.br

**Keywords:** nandrolone decanoate, skeletal muscle, physical exercise

## Abstract

*Background and Objectives:* Nandrolone decanoate (ND) is the most widely used among the anabolic androgenic steroids (AAS), synthetic substances derived from testosterone, to improve muscular and health gains associated with exercises. The AAS leads to physical performance enhancement and presents anti-aging properties, but its abuse is associated with several adverse effects. Supraphysiological doses of AAS with or without physical exercise can cause morphological and functional alterations in neuromuscular interactions. This study aims to investigate the effects of ND supraphysiological doses in neuromuscular interactions, focusing on the soleus muscle and its neuromuscular junctions (NMJs) in rats, associated or not with physical exercise. *Materials and Methods:* Forty male Sprague Dawley rats were divided into four groups: sedentary and exercised groups, with or without ND at the dose of 10 mg/kg/week. The animals were treated for eight weeks, with intramuscular injections, and the soleus muscle was collected for morphological analyses. *Results:* The supraphysiological doses of ND in the sedentary group caused muscle degeneration, evidenced by splitting fibers, clusters of small fibers, irregular myofibrils, altered sarcomeres, an increase in collagen deposition and in the number of type I muscle fibers (slow-twitch) and central nuclei, as well as a decrease in fibers with peripheral nuclei. On the other hand, in the ND exercise group, there was an increase in the NMJs diameter with scattering of its acetylcholine receptors, although no major morphological changes were found in the skeletal muscle. Thus, the alterations caused by ND in sedentary rats were partially reversed by physical exercise. *Conclusions:* The supraphysiological ND exposure in the sedentary rats promoted an increase in muscle oxidative pattern and adverse morphological alterations in skeletal muscle, resulting from damage or post-injury regeneration. In the ND-exercised rats, no major morphological changes were found. Thus, the physical exercise partially reversed the alterations caused by ND in sedentary rats.

## 1. Introduction

Anabolic androgenic steroids (AAS) are synthetic substances derived from testosterone that have physical performance-enhancing and anti-aging properties [1,2]. Athletes and bodybuilders commonly use AAS to promote growth and increase the strength of skeletal muscles [3] or for aesthetic reasons, with Nandrolone decanoate (ND) being one of the most used AAS alongside testosterone, methandienone, and methenolone [4,5,6]. These substances increase the number of satellite cells per muscle fiber, thereby accelerating muscle growth and development [7].

The use of ND is mainly associated with the practice of high-intensity and short-duration physical exercise, with weight overload exceeding the muscular aerobic capacity, due to its anabolic properties and its capacity to increase tolerance to exercise by making the muscles more capable of resisting overload [6,8,9].

Physical exercise is one of the major factors being studied concerning the skeletal muscle plasticity process [10,11,12]. It can modulate muscle fibers [13,14], and play a crucial role in metabolic overcompensation and meeting the body’s needs to improve physical performance [15,16,17]. Muscle fibers are gradually recruited according to their specific characteristics in response to exercise, with type I fibers (slow-twitch contraction) being efficient in high-resistance aerobic exercises due to their high ATP production with the presence of oxygen, and type II fibers (fast-twitch contraction) responding better to exercise performed without oxygen. However, type II fibers’ metabolism produces intramuscular lactate, which can cause early fatigue and lead to a reduction in exercise tolerance [18].

Physical exercise can also affect the nervous system by directly impacting the peripheral nervous system and neuromuscular junctions (NMJs) [19,20,21]. NMJs are extremely sensitive to exercise-induced adaptive responses, including an increase or decrease in activity, and structural changes such as nerve terminal branching [22]. Physical exercise can also cause an increase in motor endplate area and the number of nicotinic acetylcholine receptors (nAChRs) [23,24], indicating that it may play a role in shaping the structure and function of NMJs.

While ND associated with resistance exercise has been shown to improve physical performance and muscle mass gains, its abuse can lead to side effects as well as somatic consequences, including effects in cardiac and skeletal muscles such as an increase in extracellular collagen production, interstitial fibrosis, also changing the vascularization which impairs the angiogenesis, and hypertrophic myopathy [6,9,25,26]. Also, the toxic effect of ND on the NMJ has already been demonstrated, with a reduction in the safety margin of synaptic transmission in sedentary rats [27]. 

Given the high plasticity of skeletal muscles and NMJs in response to physical exercise and the increasing prevalence of indiscriminate use and abuse of AAS among adults and adolescents [6,28,29,30], it is important to investigate the effects of ND supraphysiological doses on the soleus muscle and NMJs in rats, both in the presence and absence of physical exercise.

## 2. Materials and Methods

### 2.1. Animals and Experimental Design

Forty male Sprague Dawley rats (130 days old, 400–500 g) acquired from the Multidisciplinary Center for Biological Research (CEMIB) of the University of Campinas (Unicamp) were kept under appropriate conditions in the bioterium of the Division of Anatomy, Department of Structural and Functional Biology, Institute of Biosciences of Botucatu (IBB/Unesp), State of São Paulo, Brazil. This study was approved by the Ethics Committee on Animal Use from Unesp (CEUA/IBB/Unesp, protocol 448/14).

The animals were distributed into 4 experimental groups (*n* = 10/group): sedentary (S); sedentary with Nandrolone (NDS); exercise (E) and exercise with Nandrolone (NDE).

### 2.2. Experimental Protocol

In the NDE and NDS groups, intramuscular injections of Nandrolone Decanoate were administered (Deca Durabolin^®^) diluted in propylene glycol vehicle at a dose of 10 mg/kg/week, twice a week (5 mg/kg/each time) for 8 weeks [31]. The chosen ND dosage (10 mg/kg/week) mimics, in rodents, human exposure to excessive consumption. Although this corresponds to a high and abusive dose, ranging from 10 to 100 times higher than the human therapeutic dose, it is commonly used by athletes indiscriminately to gain weight and muscle mass quickly [8,25,26,31,32,33,34,35]. The S and E groups were given intramuscular injections of propylene glycol vehicle (0.2 mL/kg), twice a week. The period of eight weeks for the treatment was selected based on similar experimental studies that have reported observable effects of ND exposure on tissue changes, including male reproductive organs, and skeletal and cardiac muscles [8,25,31,33,34,35].

### 2.3. Resistance Physical Exercise by Jumping in a Liquid Medium

The treatment with ND and analysis of physical exercise began when the animals reached 56 days of age. The E and NDE groups were subjected to jumping sessions in a Polyvinyl Chloride cylinder containing water with a depth of 38 cm at a temperature of 30 °C [33,36]. During the first week, rats in the exercise groups underwent an adaptation period to the exercise in the liquid medium. After this, from the second week on, the animals were subjected to 4 series of 10 jumps, with 60 s intervals, three times a week. The weight overload was progressively increased based on the animal’s weight, with 50% in the 2nd and 3rd weeks, 60% in the 4th and 5th weeks, and 70% in the 6th, 7th, and 8th weeks (Table 1). The weight overload was placed on the ventral chest using a vest and after each exercise session, all animals were dried with cotton towels and kept warm for 30 min. 

Throughout the physical exercise period, the sedentary animals (S and NDS) were not subjected to jumping sessions and were instead placed in a box with shallow water at a temperature of 30 °C.

### 2.4. Material Processing

Two days after the last jumping session, all animals were weighed and then euthanized. Five animals from each experimental group were euthanized using a CO_2_ chamber, and the other five animals from each group were anesthetized through intraperitoneal injection of Ketamine (Dopalen, 90 mg/kg) and Xylazine (Anasedan, 10 mg/kg), and subjected for transcardiac perfusion to analysis of NMJ by confocal microscopy.

The soleus muscles were dissected, removed, weighed, and processed according to the protocols below. The soleus muscle was chosen for this study considering that it is a postural muscle of utmost importance, whose function is associated with maintaining stability and locomotion [37]. Its oxidative nature, rich in mitochondria, has been demonstrated as crucial for performing endurance exercises. Further, it offers greater convenience for locating and visualizing NMJs, particularly in the middle third where the motor point is evident.

### 2.5. Morphological and Morphometric Analysis of NMJ

The muscles were reduced to the middle thirds (regions containing the motor point) and sectioned longitudinally into 4 pieces using a metal sheet. The slices were submitted to a nonspecific esterase reaction [38] to mark the NMJs. The images of 50 NMJs were used for maximum diameter morphometric analysis. The measurements were analyzed with ImageJ software (version 1.53).

### 2.6. Ultrastructural Analysis of NMJ

The soleus muscle portions containing the motor point were cut, submerged into Karnovsky solution (4% paraformaldehyde and 2.5% glutaraldehyde, in phosphate-buffered saline—PBS, 0.1 M, pH 7.4) and subjected to the routine technique for Transmission Electron Microscopy (TEM) of the Electron Microscopy Center (CME) of the IBB/Unesp. The tissues were included in such a way that longitudinal muscle sections were obtained for the identification of the muscle fibers and the NMJs. The ultrathin sections obtained were examined and documented using a Transmission Electron Microscope (FEI/Philips CM100 model).

### 2.7. Analysis of Acetylcholine Receptors of NMJ by Confocal Microscopy

Five animals from each experimental group were submitted to transcardiac perfusion with PBS and then fixed in 4% paraformaldehyde (in PBS, 0.1 M, pH 7.4). The caudal vena cava was severed at the level of the right auricle to drain blood and the excess infusion fluid. The soleus muscle was collected, reduced to the middle third (region containing the motor point), and submitted to the protocol for nAChRs labeling. After post-fixation for 15 min, the muscle fragments were flushed several times with PBS. To inactivate the fixative, the fragments were incubated in 0.1 M glycine for 20 min in an orbital shaker. After flushing with PBS, they were incubated with 1% collagenase (Sigma Type I C-0130) for 30 min on the shaker to detach the connective tissue from the muscle. The muscles were flushed with PBS and incubated with Triton X-100 4% (Sigma T9284, in PBS) for 1 h to attain permeabilization of the muscle fibers. They were subsequently flushed with PBS and the nAChRs were labeled with alpha-bungarotoxin conjugated to rhodamine (Tetramethylrhodamine α-Bungarotoxin, Invitrogen—Thermo Fisher Scientific, T1175, 1:100 in PBS) in the shaker at room temperature for 60 min. The muscles were flushed with PBS, and the slides were mounted using VECTASHIELD^®^ mounting medium (Vector Laboratories, Burlingame, CA, USA) and photographed using a Laser Scanning Confocal Microscope (Leica TCS-5-SPE) of the CME/IBB/Unesp.

### 2.8. Morphological and Morphometric Analysis of Muscle Fibers

The ends of muscle from each animal were coated with neutral talc, frozen in liquid nitrogen, and stored in a freezer at −80 °C. Histological sections were obtained on a cryostat (4 µm, Leica CM 1800). Four slides were obtained for each animal. One slide was stained with hematoxylin and eosin (HE), and photographed on an Olympus BX41 microscope (20×). Around 200 muscle fibers selected from 3–4 random fields were used for the general morphological analysis of the muscles and counting of the fibers with central and peripheral nuclei, using ImageJ software (version 1.53).

### 2.9. Intramuscular Collagen Analysis

Another slide was stained with Picrosirius Red, the collagen in red, and the muscle fibers in yellow. To quantify intramuscular collagen, about 6 random images (20×) were obtained from each animal of each experimental group. The percentage (%) of collagen was automatically obtained using Leica QWin software (Leica Microsystems, Wetzlar, Germany). Images with polarized light were also obtained from the same slide to complement the analysis of the morphology of collagen fibers.

### 2.10. Immunohistochemistry Analysis (Fast and Slow-Twitch Fibers)

The remaining slides were subjected to immunohistochemistry using the Strept AB Complex/HRP immunoperoxidase method for specific primary antibodies: Fast Anti-myosin (WB-MYHCf, Novocastra, 1:160) and Slow Anti-myosin (WB-MYHCs, Novocastra, 1:120). Histofine1 (Multi-Nichirei) was used as the secondary antibody. The specific staining was revealed by the chromogenic substrate DAB (1:50). After identifying the types of muscle fibers (in cross-sectional slices), 5–6 fields were photographed per slide, to obtain about 200 fibers. The types of fibers were counted, their area measured, and the frequency was calculated using ImageJ software (version 1.53).

### 2.11. Statistical Analysis

The weight of the animals and the soleus muscle, maximum diameter of NMJs, and percentage of collagen were analyzed by the one-way ANOVA test followed by a post-test of multiple comparisons of Tukey [39]. The number of fibers with central and peripheral nuclei and the numbers of slow- and fast-twitch fibers were analyzed by the Kruskal–Wallis test followed by a post-test of multiple comparisons of Dunn [39]. The frequency of slow- and fast-twitch fibers was analyzed by Goodman’s association test complemented by multiple comparisons between and in the multinomial populations [39]. The results were expressed as mean and standard deviation (SD). All data were considered using the 5% significance level (*p* ≤ 0.05).

## 3. Results

### 3.1. Body and Soleus Muscle Weights

**Figure 1:**
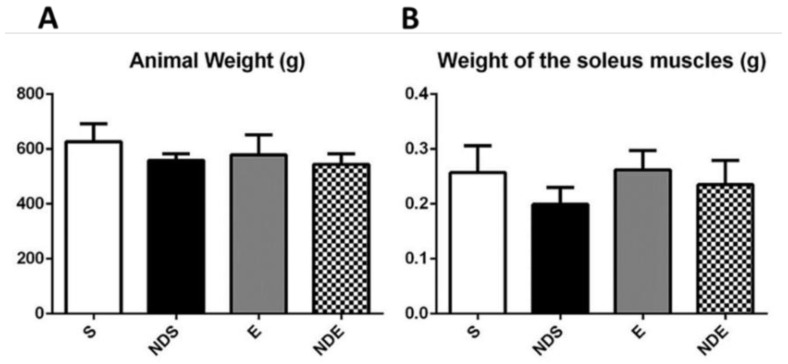
Body weight of the rats (**A**) and wet weight of the isolated soleus muscle (**B**) of all experimental groups. Values expressed as mean and SD, and analyzed by the one-way ANOVA test complemented by the test of multiple comparisons of Tukey (*p* > 0.05).

There was no statistically significant difference in the body weight of the animals and the wet weight of the muscles among all experimental groups, regardless of the ND use (Figure 1).

### 3.2. Neuromuscular Junction Results

In all groups, the NMJs were aligned transversely or obliquely to the long axis of the muscle fibers, next to the nerve branches that take axons to these endplates.

**Figure 2:**
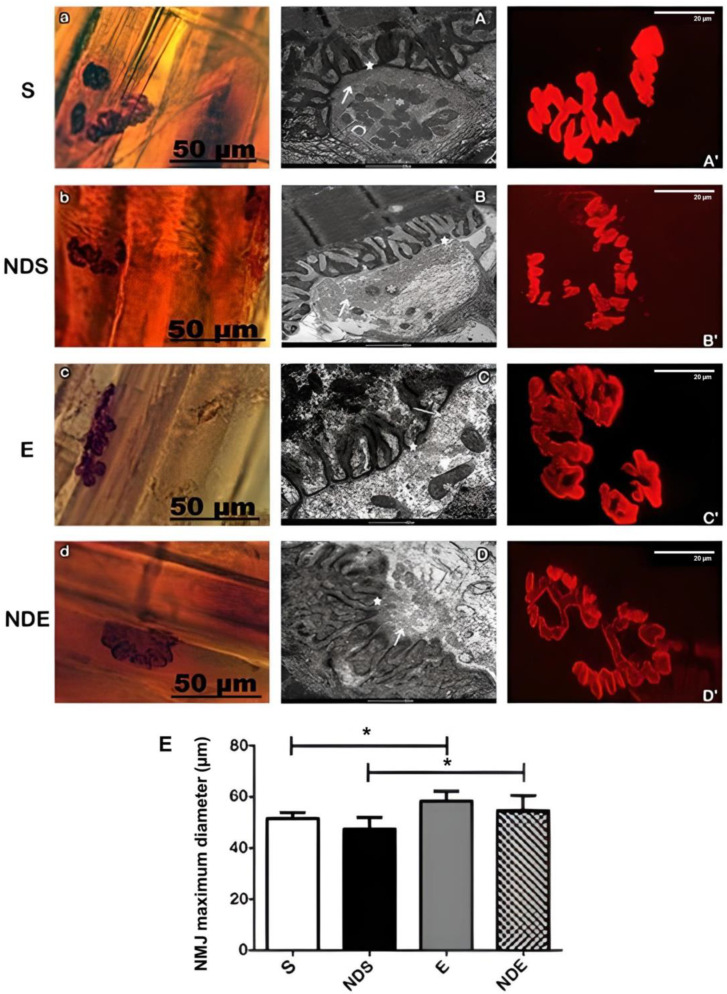
Photomicrographs of neuromuscular junctions of the soleus muscle of all experimental groups. (**a**–**d**) neuromuscular junctions labeled with nonspecific esterase. (**A**–**D**) Transmission electron microscopy of neuromuscular junctions. Mitochondria (*), synaptic vesicles (arrow), electronically dense postsynaptic region (★). (**A’**–**D’**) Confocal microscopy of acetylcholine receptors of the neuromuscular junctions labeled with alpha-bungarotoxin conjugated rhodamine. (**a**,**A**,**A’**) S group. (**b**,**B**,**B’**) NDS group. (**c**,**C**,**C’**) E group. (**d**,**D**,**D’**) NDE group. (**E**) Measurement of maximum diameter (μm) of neuromuscular junctions (NMJ) present in each experimental group. Values expressed as mean and SD and analyzed by the One-Way ANOVA test complemented by the test of multiple comparisons of Tukey (* *p* < 0.05).

These junctions were intensely branched and presented wide synaptic gutters. Variations in form (open, irregular, and compact) characterized the polymorphism of these structures but they should not be considered as morphological changes related to the studied groups (Figure 2a–d (A–D)).

By ultrastructural analysis, all the NMJs studied showed a standard morphology. They presented the axon terminals arranged in synaptic gutters as sometimes shallow or deep with variable numbers of synaptic vesicles and mitochondria (Figure 2A–D). In the presynaptic membrane, the presence of electronically denser regions was evident. Those correspond to active zones which are opposite to the apex of the junctional folds of the postsynaptic membrane (Figure 2A–D).

A mild scattering of nAChRs was observed in the physically exercised group (with or without ND exposure) (Figure 2C’,D’). This result was confirmed by NMJs morphometry (Figure 2E).

The NMJs maximum diameter analysis demonstrated that there was an increase in the exercise group, with (54.6 ± 5.9 μm) or without ND (58.4 ± 3.9 μm), compared to the sedentary group with (47.4 ± 4.6 μm) or without ND (51.6 ± 2.3 μm) (*p* < 0.05) (Figure 2E).

### 3.3. Muscle Fibers Results

**Figure 3:**
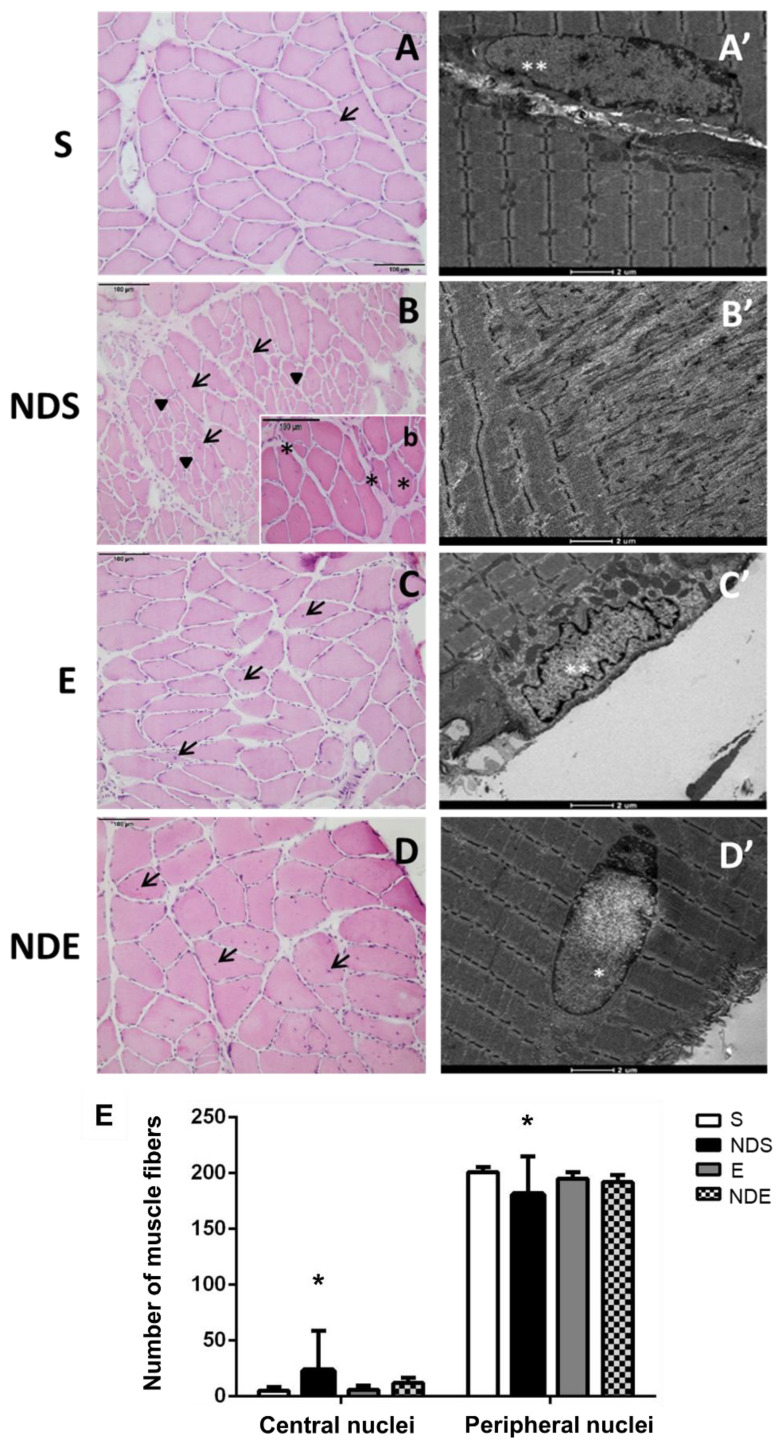
Photomicrographs of soleus muscle cross sections of all experimental groups. (**A**–**D**,**b**) HE stained. Muscle fibers with the presence of central nuclei (arrow/→), regions with small fibers with peripheral nuclei (arrowhead/►), and regions of splitting fibers (*). (**A’**–**D’**) Transmission electron microscopy of muscle fibers. Peripheral nuclei (**), central nucleus (*). (**A**,**A’**) S group. (**B**,**b**,**B’**) NDS group. (**C**,**C’**) E group. (**D**,**D’**) NDE group. (**E**) Quantification of central and peripheral nuclei. Values expressed as mean and SD, and analyzed by the Kruskal–Wallis test followed by a post-test of multiple comparisons of Dunn (* *p* < 0.05, compared to all experimental groups).

Morphological analysis was carried out by examining images obtained after HE staining and TEM use. In the S group, the fibers had a polygonal shape, peripheral nuclei, and preservation of the endomysium and perimysium (Figure 3A). The myofibrils were ultra-structurally organized in the muscle fibers forming well-defined sarcomeres with morphological features related to the different types of muscle fibers. The most frequent type found in the soleus muscle contained thick Z-lines, intermyofibrillar mitochondria, and T-tubules organized in triads (Figure 3A’).

In the NDS group, it was observed that some fibers lost their typical shape, and regions containing fibers of small diameter with peripheral nuclei were found (Figure 3B). Other regions presenting splitting fibers with central nuclei were also found (Figure 3B’). Altered myofibrils with disorganized sarcomeres were present in the TEM analysis.

In the NDE and E groups, morphology was preserved, and the fibers presented a typical shape and size (Figure 3C,D). In the NDE group, the presence of fibers with central nuclei was also detected (Figure 3D).

An increased number of central nuclei and a decreased number of peripheral nuclei of muscle fibers were found in the NDS group, compared to all experimental groups (*p* < 0.05) (Figure 3E).

### 3.4. Intramuscular Collagen Results

**Figure 4:**
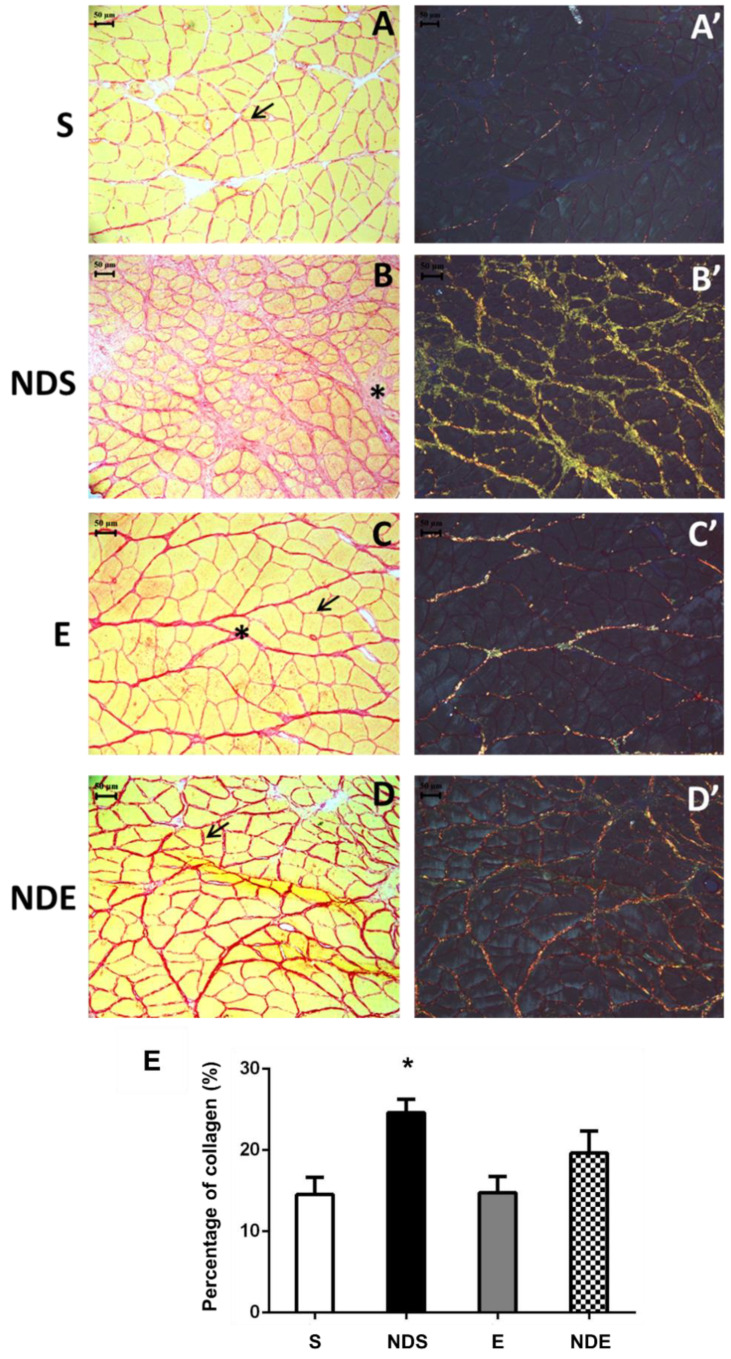
Photomicrographs of soleus muscle cross-sections stained by Picrosirius red from all experimental groups under conventional light microscopy (**A**–**D**) and polarized light microscopy (**A’**–**D’**). (**A**,**A’**) S group. (**B**,**B’**) NDS group. (**C**,**C’**) E group. (**D**,**D’**) NDE group. Endomysium (arrow/→), perimysium (*). (**E**) Graph of percentage of the collagen areas of all experimental groups. Values expressed as mean and SD and analyzed by the one-way ANOVA test complemented by the test of multiple comparisons of Tukey (* *p* < 0.05, compared to all experimental groups).

The collagen fibers present in the endomysium and perimysium were visualized by Picrosirius Red staining. Collagen fibers were stained in red while muscle fibers in yellow (Figure 4A–D). This analysis was conducted using conventional and polarized light optical microscopes (Figure 4A’–D’). The morphological analysis demonstrated increased red staining in the NDS and NDE groups, suggesting increased collagen, mainly in the NDS group (Figure 4B,D). NDS group animals presented an increased deposition of collagen fibers in the perimysium region (Figure 4B). This observation was confirmed by polarized light, the fibers were clustered and strongly birefringent, occupying mainly the region of the perimysium. Their birefringence ranged from yellow to green, suggesting the presence of collagen fibers type III (newly synthesized) (Figure 4B’). In the other groups, a small amount of deposition of collagen fibers was detected, and under the polarized light; they showed red tones (suggestive of the presence of type I collagen—mature collagen) (Figure 4A,A’,C,C’).

The quantification of collagen areas (percentage) demonstrated statistically the observations in morphology described above. The NDS group had an increase in the percentage of the collagen area compared to all experimental groups (*p* < 0.05) (Figure 4E).

### 3.5. Immunohistochemistry Results

**Figure 5:**
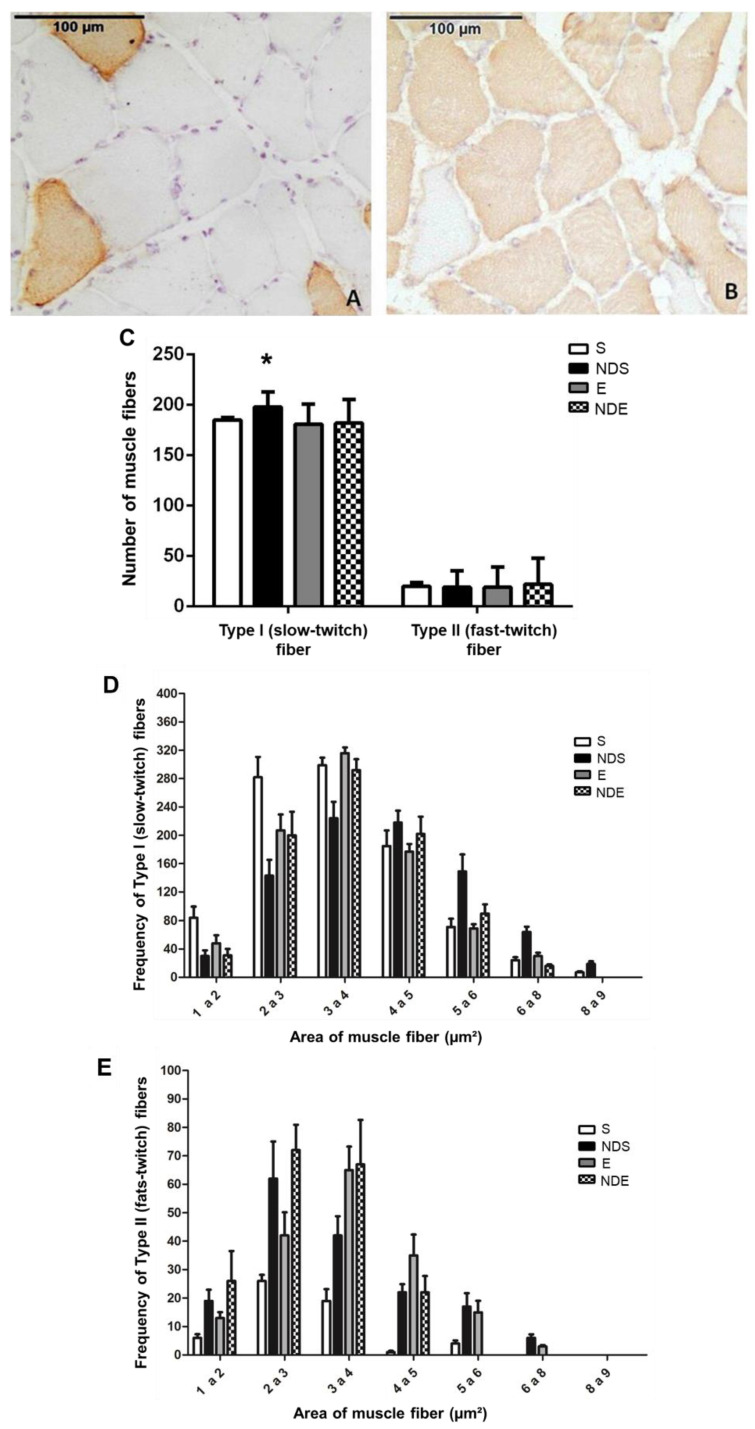
Photomicrographs of soleus muscle cross-sections of the NDS group immunostained for type II/fast-twitch fibers (**A**) and type I/slow-twitch (**B**) fibers. (**C**) Quantification of slow-twitch (type I) and fast-twitch (type II) fibers according to the experimental groups. Values expressed as mean and SD and analyzed by the Kruskal–Wallis test followed by a post-test of multiple comparisons of Dunn (* *p* < 0.05; compared to all experimental groups). (**D**,**E**) Frequency of areas of type I/slow-twitch (**D**) and type II/fast-twitch (**E**) fibers according to classes (thousand—µm^2^). Values expressed as mean and SD, and analyzed by the Goodman’s association test complemented by multiple comparisons between and in the multinomial populations (*p* > 0.05).

The immunohistochemistry staining showed the type I fibers (slow-twitch) strongly stained in brown (Figure 5B), where the antibody for slow myosin was used, whereas the type II fibers (fast-twitch) did not react to the chromogen (Figure 5A). On the slides, where fast myosin antibody was used, the type II fibers (fast-twitch) were stained, whereas type I fibers (slow-twitch) were not (Figure 5A,B).

This analysis detected a greater quantity of type I muscle fibers, as there was a predominant expression of the slow myosin heavy chain in relation to the fast myosin (type II fibers). This predominance was present in all experimental groups, being the standard for the soleus muscle—predominantly oxidative (Figure 5A,B).

In the NDS group, a statistically significant increase in type I fibers (slow-twitch) was found compared to all experimental groups (*p* < 0.05) (Figure 5C). The amount of type II fibers (fast-twitch) remained stable about the parameters analyzed (Figure 5C).

A class representation of the fiber areas was created to better understand the modulation of fast-twitch and slow-twitch fibers as a response to the treatments (exercise and use of ND), facilitating the comparison among groups. Respecting the classification in slow-twitch and fast-twitch fibers, the frequency of the areas was sorted as follows: 1–2 and 2–3 thousand μm^2^—smaller classes; 3–4 and 4–5 thousand μm^2^—intermediate size classes; fibers with a diameter greater than 5000 μm^2^—larger classes (Figure 5D,E).

Regarding the slow-twitch fibers in the S group, there was a predominance of fibers ranging in size from 2–3 and 3–4 thousand μm^2^. In the NDS group, the fibers with areas 3–4 and 4–5 thousand μm^2^ were predominant. Among the exercised animals, there was slow-twitch fiber predominance with a 3–4 thousand μm^2^ area (Figure 5D).

Observing the area of fast-twitch fibers, small class fibers were predominant with diameters of 2–3 thousand μm^2^ in both groups of sedentary animals. In the E group, the predominant class was 3–4 thousand μm^2^ (intermediate class). The animals of the NDE group, fibers with 2–3 and 3–4 thousand μm^2^ areas predominated (Figure 5E).

## 4. Discussion

The purpose of this study was to investigate the effects of supraphysiological doses of ND on muscle fibers and NMJs of adult rats’ soleus muscles, with or without physical exercise. As the incidence of deliberate human exposure to steroids increases, investigations into the effects and risks of ND, particularly in supraphysiological doses without medical prescription, have become more common across the scientific community [3,6,25,40,41].

Our study found that the use of ND in supraphysiological doses without physical exercise led to changes in muscle fibers, including focal lesions and splitting fibers, as well as an increase in the number of central nuclei and the percentage of collagen and type I fibers.

Melo Neto et al. [35] investigated the same dose of ND (10 mg/kg/week) in association with physical exercise on prostate microvasculature and observed weight reduction in the animals that received the drug. However, in our study, we did not find a difference in the weight of the animals or of the soleus muscle, but a trend toward weight reduction was observed in the NDS and NDE groups. The mechanisms involved in the reduction of body weight are related to decreased appetite and increased lipid oxidation, amino acid uptake, and protein synthesis amelioration caused by excessive doses of AAS [42,43]. Another study by Horstman et al. [44] found that the administration of ND during a short period of leg immobilization in humans did not preserve skeletal muscle mass and strength.

Regarding the NMJs, no considerable morphological changes were observed. However, morphometric analysis showed an increase in the maximum diameter in the animals that underwent physical exercise compared to the sedentary animals. NMJs are highly plastic structures, whose diameter can remodel rapidly in response to different stimuli, including denervation [45,46,47,48], aging [33], or drug therapies [49]. Primary alterations in the NMJ can directly generate a functional loss in several diseases, resulting in skeletal muscle weakness and increased fatigue [50]. Exercise induces hypertrophy of NMJs, whereas decreased physical activity causes degenerative changes in NMJs [21]. These changes likely occur as part of an adaptive response of the neuromuscular system to stimuli, potentially enhancing neuromuscular efficiency or leading to hypertrophy, which is a recognized outcome of exercise [51]. Similar results were reported in rats that followed a seven-week resistance exercise program, where the animals exhibited increased total area and perimeter of the NMJs [35,52]. It is well known that muscle hypertrophy promoted by physical exercise is followed by a direct increase in the number of NMJs associated with those muscle fibers [35].

We observed alterations in muscle fiber morphology, with regions containing clusters of small and splitting fibers, only in the ND groups, but with a higher incidence in the NDS group. In the NDE group, the muscle fibers had a morphology closer to normality; thus, the exercise may have inhibited this action of the synthetic steroid. Muscle fiber branching and splitting are typically observed during muscle adaptation, mainly hypertrophy, as a consequence of damage and regeneration via satellite cells, often associated with resistance exercise [53]. These alterations have also been demonstrated in animals exposed to AAS [54], and subjected to various physical exercise protocols [55,56]. 

Studies have shown that chronic ND administration can cause apoptotic effects in adult rat ventricular myocytes [57] and in the rodent brain and kidney [58,59], and lead to irreversibility by destroying heart tissue [60]. This apoptotic outcome could be triggered by an increase in inflammatory cytokines such as TNF-α, which promotes apoptosis through interaction with its TNFR1 membrane-bound receptor or participates in mitochondrial membrane disturbances with the subsequent release of cytochrome C and cell death [58]. The consequences of AAS abuse are diverse and depend on dosage, type, frequency, and usage model [3,58]. However, it is well known that high doses can cause acute and/or chronic adverse side effects in almost all major tissues and organs [3,61,62].

Our study found that the NDS group had a higher presence of central nuclei compared to all other experimental groups. Central nuclei are normally present in regenerating muscle fibers after muscle injury [63]. This alteration has also been observed in association with AAS use [64]. Additionally, the NDS group exhibited an increased amount of collagen fibers compared to all other experimental groups. This finding has also been reported in heart muscle and kidney, where the increase in collagen deposition after ND use was considered one of the changes resulting from abnormal organ hypertrophy [65]. It has been suggested that anabolic steroids can stimulate the synthesis of collagen III [66]. Under polarized light, the deposition of collagen fibers in the NDS group appeared to represent newly synthesized collagen-III fibers (green color), indicating a post-injury regenerative process in the muscle tissue [67].

The E and NDE groups showed an increase in type I collagen fibers, which was more evident in the NDE group, as evidenced by the presence of more reddish fibers under polarized light. This finding has also been reported in heart muscle with the use of AAS associated with exercises, and it has been suggested as a possible aspect of abnormal heart hypertrophy [68].

A previous study investigated the effect of ND on quadriceps muscle [26]. The study found mild skeletal muscle hypertrophy in sedentary Wistar rats that received ND for 4 weeks with training, along with an increase in collagen deposition. Although the exact mechanism of ND-induced fibrosis is not yet fully understood, it is suggested that oxidative stress and inflammatory cytokines may trigger proliferative and fibrotic pathways that lead to increased collagen deposition, as also observed in cardiac muscle tissue [69,70].

Immunohistochemistry and its quantification revealed an increase in type I fibers (slow-twitch) in NDS animals compared to the S group, which is in line with a previous study [54]. This previous study showed that an increase in oxidative fibers after mesterolone use caused an increase in the number of mitochondria, thus enhancing fatigue resistance. Another study [71] that tested a resistance exercise protocol with insufficient recovery time also found an increase in type I and type IIB fibers in the plantar muscle.

Moreover, an increase was observed in the area of oxidative fibers (type I) in groups that received ND compared to S, while no alteration in the area of this type was observed in NDE and E animals. This increase may be attributed to the anabolic profile of the AAS, as previously described [72].

Among the glycolytic fibers, no changes were observed in the NDS group, which maintained the same predominance of class as seen in the S group. However, in the NDE and E groups, there was an increase in the area of type II fibers compared to the S group. These results may be attributed to the catabolic counterpoint that occurs as a consequence of resistance exercise, which counteracts the anabolic effect of AAS [72].

The increasing prevalence of AAS use (and abuse), especially among young individuals and bodybuilders, is a matter of growing concern in light of the findings presented here. This increasing use can be attributed to a growing preoccupation with body aesthetics, seeking to meet beauty standards and the current ideal of physical perfection, which reflects the high standards of performance, health, and beauty required by contemporary society. To attain these standards, young people and bodybuilders are increasingly using and promoting chemical substances and hormonal therapies which intervene in biological processes, such as testosterone, to tone the body in the quest for perfection [6,28,73]. Our results, concerning AAS abuse in a sedentary state, clarify the potential risks associated with it, both in terms of skeletal muscle health and overall well-being. Over time, these detrimental alterations can lead to irreversible impairments in quality of life. It is crucial for health institutions to raise awareness of the potential health hazards linked to AAS abuse.

It is important to note that our results pertain to a particular type of exercise protocol combined with the use of supraphysiological doses of ND over an 8-week period. Therefore, it is crucial to emphasize that deviations in the exercise protocol, type of AAS, route of drug administration, dosage, animal model, and treatment duration could yield different outcomes. The limitation of this study was to focus only on an oxidative muscle. The comparison among muscles with varying metabolic profiles, including apoptosis markers, could provide further insights into broader aspects of muscle morphology alterations.

## 5. Conclusions

The findings of this study indicate that the use of ND without physical exercise did not promote gains in muscle and body mass or muscle weight, but induced adverse morphological changes in the soleus muscle, such as splitting fibers, irregular myofibrils, altered sarcomeres, increase in the number of central nuclei and type I muscle fibers (slow-twitch), and increase in collagen deposition. Although major alterations have not yet been found regarding the NMJs, further studies are needed to investigate AAS use in a sedentary state.

The supraphysiological ND exposure in the sedentary rats promoted an increase in muscle oxidative pattern and adverse morphological alterations in skeletal muscle, resulting from damage or post-injury regeneration. However, in the ND-exercised rats, no major morphological changes were found. Thus, the physical exercise partially reversed the alterations caused by ND in sedentary rats.

## Figures and Tables

**Table 1:** Exercise protocol according to the day and body weight (%).

Exercise Day	Exercise-Overload (% Body Weight)
1st	2 series of 5 jumps (50%)
2nd	3 series of 5 jumps (50%)
3rd	4 series of 5 jumps (50%)
4th	4 series of 7 jumps (50%)
5th	4 series of 9 jumps (50%)
6th to 20th	4 series of 10 jumps (50%)
21th to 35th	4 series of 10 jumps (60%)
36th to 54th	4 series of 10 jumps (70%)

## Data Availability

The datasets generated or analyzed during the current research are available from the corresponding author upon reasonable request.
